# Supplementary material for: Are carbon emissions trading and green financial instruments synergistic? -Comprehensive quantitative research based on content analysis
Source: PLoS One. 2024 Mar 7;19(3):e0298601. doi: 10.1371/journal.pone.0298601 (PMC10919601; doi:10.1371/journal.pone.0298601)
Supplement: S1 Data — (ZIP) [file pone.0298601.s001.zip › Data/地级市Z_F工作报告.zip!/╝¬┴╓/┴╔╘┤╩╨/└┤╫╘╣1⁄2╓┌║┼í╛┬φ┐╦╩2╛▌═°í┐_2014.docx]

各位代表：

　　现在，我代表市政府，向大会作工作报告，请予审议，并请市政协各位委员提出意见。

　　一、2013年工作回顾

　　过去的一年，在省委、省政府和市委的正确领导下，全市上下团结一心，积极应对挑战，奋力攻坚克难，我市全面转型迈出新步伐，各项事业取得新进展。

　　经济持续健康发展。预计地区生产总值达到700亿元，可比增长9%。地方级财政收入完成25.5亿元，增长7%。固定资产投资增长22.8%，社会消费品零售总额增长12%，外贸进出口总额增长22.9%。节能减排完成省计划。农村经济稳定发展。粮食产量达到46.7亿斤。新增市级以上农业产业化龙头企业15户。金州、安恕、拉拉河等现代农业园区建设稳步推进。东丰县在全省县域发展综合考评中由18位晋升到14位。工业主导地位进一步巩固。规上工业总产值实现1185亿元，增长15%。规上工业企业达到306户，其中产值超亿元企业205户。服务业发展呈现新变化。现代物流、信息服务等新型业态正在形成。辽源电子商务园区启动运行。第三产业增加值达到226亿元，可比增长11.5%。项目建设扎实推进。实施3000万元以上项目420个，其中新开工203个、竣工156个。利源轨道车体深加工、聚源重型矿山设备、元隆达内高压成型等项目投产。园区建设步伐加快。辽源经济开发区启动升级转型，在全省综合评比中位列第五名，为晋升国家级开发区奠定了坚实基础。县区工业集中区承载功能不断提升。科技创新成效明显。成立铝合金钻具材料研究所和吉林省化药、针纺产业技术创新战略联盟。新认定3个中国驰名商标。我市获批建设省级可持续发展实验区和省级创新型试点市。民营经济更趋活跃。建立企业孵化器13个，孵化企业237户。民营经济增加值占GDP比重达到62%，主营业务收入等重要指标增幅保持全省领先。

　　特色城镇化扎实推进。城乡建设迈出新步伐。南部新城建设稳步实施。东丰县西城区开发步入良性循环。东辽县新城区建设加快推进。金州乡被确定为全省首批新型城镇化试点乡镇。基础设施得到新提升。伊开高速一期完成基础工程，辽长铁路开工建设，集锡线、营西线、烟辽线等大中修工程完工，新建农村水泥路205公里。杨木水库输水复线、东辽河干流防洪、北城区供水改造工程进展顺利。市区九路三桥和26条巷路改造竣工。北部热源厂一期投入使用。新增管道燃气用户7500户。城区24小时供水率提高15个百分点。五城联创步入新阶段。完成省级生态城创建任务，国家级卫生城、园林城创建工作全面启动，国家级文明城、环保模范城建设有序推进。城区绿化率提高到40%。主要街路实现12小时保洁。累计拆除违法建筑8.6万平方米，城市管理逐步规范。智慧城市建设实现新突破。我市成功列入全国首批智慧城市试点。软件及电子产品制造企业达到26户。云计算中心、应急监控指挥中心、地理信息系统投入使用。智慧城管、智慧交通、市民一卡通等项目建成。智慧城市配套设备生产制造基地投产。获得智慧城市领军城市等多项殊荣。生态建设取得新进展。水源地保护一期工程、仙人河（半截河）污水管网改造完工，矿山湿地公园建设有序推进。累计清收林地2.18万公顷。解决2.75万农村人口饮水安全问题。节水型社会建设试点工作通过国家验收。鴜鹭湖、聚龙潭、龙首山魁星楼被评定为国家3A级旅游景区。

　　改革开放不断深入。重点领域和关键环节改革进一步深化。组建市容环境管理局，理顺水务管理体制，完成部分政府部门机构改革。减少行政审批项目354个，审批提效23%。启动营改增试点。强化政府债务、财政资金和国有资产管理，清收历年拖欠财政资金9.5亿元。新成立2户担保公司和7个行业互保池。东丰县土地收益保证贷款试点取得实效。银行存贷款余额分别增长15%和22%，融资担保能力居全省第四位。积极推进土地收储交易制度改革、城乡建设用地增减挂钩试点和城乡土地综合整治，我市被国土资源部评为全国国土资源节约集约模范市。煤矿企业兼并重组工作积极推进。医药卫生体制改革走在全省前列。招商引资进一步强化。与美国微软、新加坡佳通、韩国韩华、沈阳中农置业、天津力神、吉林修正等大企业达成合作意向，百家易贷融资平台、台湾大润发商业综合体、速能达二维码应用、辽宁曙光汽车扭力梁总成等40个项目签约落地。引进域外资金475亿元，增长13%；实际利用外资2.5亿美元，增长25%。

　　民生状况不断改善。46件惠民实事全面完成，全市民生投入达到47.5亿元。预计城镇居民人均可支配收入和农民人均纯收入达到23800元和10529元，分别增长12%和15%。在建保障房294万平方米，回迁居民1.7万户。综合整治住宅小区17个。实施暖房子改造192.6万平方米。建立创业促就业基地36个，发放小额担保贷款1.85亿元。新增城镇就业4万人，城镇登记失业率控制在3.56%以内。城镇职工养老、失业保险待遇支出13亿元，新农保、城居保参保率100%，启动厂办大集体改革和国企改制失业金发放工作。农村标准化卫生室达到149个，基层医疗机构全面推行基本药物制度，药价平均下降30%。建立居民大病救治体系，城乡低保、农村五保供养标准处于全省中上游水平。一批民办养老机构、社区老人日间照料中心投入使用。新改扩建标准化幼儿园20所，教育资源配置趋向均衡。我市荣获全省人口计生考核综合先进奖第一名。成立文化产品展示交易中心。群众性文化活动蓬勃开展。征集评选出市树、市花、市标。十七届省运会筹备工作进展顺利。

　　社会保持稳定和谐。推进平安辽源建设，我市连续三年荣获全国见义勇为城市奖，群众安全感和政法机关满意度排名全省第一位，十位一体道路交通事故快速处理机制被评为全国加强和创新社会管理十佳案例。坚守安全发展红线，整改各类安全隐患14540处，其中重点隐患20处，关闭退出地方煤矿10户，重点高危行业和规模以上工贸企业推行安全生产标准化，我市荣获全国安全生产月活动先进单位称号，连续五年获省政府安全生产目标责任制考核一等奖。建立食品药品安全四级监管网络，查处各类食品药品安全案件1002件。抵御罕见洪涝灾害取得胜利，灾后重建任务全面完成。连续29年无重大森林火灾。统筹推进民族宗教、外事侨务、档案、史志、气象、地震、人防工作和老龄、残疾人等各项事业。军政军民团结不断巩固。

　　政府自身建设得到加强。自觉接受人大法律监督、工作监督和政协民主监督，广泛听取社情民意，办理人大代表建议143件、政协委员提案94件。进一步理顺政府职能，强化行政效能，科学民主决策，狠抓工作落实，不断提升公信力和执行力。深入开展行政监察和审计监督，严肃查处违法违纪案件，着力整治发展软环境。严格执行中央八项规定，坚决纠正四风一顽症， 不断改进会风文风，扎实推进治理超标配车、清退超标办公用房等工作，三公经费大幅下降。

　　各位代表，过去的一年，宏观经济形势复杂多变，我们在挑战增多、矛盾加大、困难和压力超出预期的情况下，取得了十分不易的成绩。这得益于市委的正确领导，得益于市人大、市政协的监督支持，得益于各县区、各部门、各方面的艰苦努力，更得益于广大干部群众的积极工作、广泛参与、大力支持。在此，我代表市政府，向奋战在各条战线上的广大劳动者，向关心支持政府工作的人大代表、政协委员、各民主党派、工商联、人民团体和无党派人士，向老领导、老同志，向热心参与辽源发展建设的驻辽部队、中省直单位、海内外朋友致以崇高的敬意和衷心的感谢！

　　我们更清醒地认识到，辽源经济社会发展还面临很多矛盾和问题。受宏观环境影响，经济发展速度放缓；减收增支因素增多，政府债务风险加大；项目谋划和储备不足，特别是落地项目少、投资强度低、投产达产慢等问题比较突出；产业规划、园区规划、城市规划仍需加强；融资、土地、房屋征收、软环境建设等方面难题有待深入破解；有的部门和公职人员责任意识、创新精神、服务水平、工作作风亟待强化和提升。对此，市政府将采取有效措施切实加以解决。

　　二、2014年工作安排

　　2014年，对于辽源转型发展至关重要，我们面临的形势更为复杂，任务异常艰巨，挑战和机遇也前所未有。市政府要深入贯彻党的十八届三中全会、中央经济工作会议、省委十届三次全会、全省经济工作会议和市委六届三次全会精神，把握全面深化改革总要求和稳中求进总基调，坚持工业化、信息化、城镇化、农业现代化四化统筹，推进创新发展、统筹发展、绿色发展、开放发展、安全发展。突出总量扩张、产业升级、改革开放、城镇化建设、县域发展、改善民生、生态建设、社会治理等重点任务，加快经济、政治、文化、社会、生态文明建设，奋力开创辽源转型发展新局面。

　　主要预期目标是，地区生产总值可比增长9%，地方级财政收入力争与经济增长同步；固定资产投资增长17%左右；社会消费品零售总额增长13%；城镇居民人均可支配收入、农民人均纯收入增长12%和14%左右；居民消费价格指数涨幅控制在3.5%左右；城镇登记失业率控制在3.9%以内；城镇化率提高1个百分点；节能减排完成省计划。工作中有些指标力争完成得更好一些。

　　（一）保持经济稳健运行。把握转变发展方式主线，确保经济增长处于合理区间，切实提高发展质量和效益。强化投资拉动。围绕保障性安居工程、重大基础设施、城镇化建设、社会事业、生态环保等重点领域，用足用好资源衰退型城市可持续发展政策，争取得到国家更多支持。立足打造特色产业集群，充分发挥企业主体作用，强力推进立市型、税源型项目。更加注重规划、环保、安全、节能监管和节约集约用地。强化项目带动。组织实施亿元以上项目超过220个, 其中新开工项目不少于77个、10亿元以上项目不少于33个。全力推进麦达斯板带箔材、金翼循环经济产业园、均胜汽车电子零部件、中意科技产业园、方大锻造锻压生产线、凯旋王国创意园、辽河北岸城市综合体等重点项目。谋划储备重大工业、服务业项目200个以上。强化创新驱动。引导企业参与创新成果研发和进入产业技术创新联盟，探索与省内高校、科研院所共建科技成果转化中心，加快组建元隆达、中医院院士工作站。鼓励引导企业创造自主品牌。积极争取列入国家质量强市示范城市，适时申报国家级可持续发展实验区。注重发挥消费促动作用。制定完善商业发展规划，出台促进消费政策，拉动本地消费，吸引外部消费。继续实施万村千乡市场工程，完善农村商业连锁经营和统一配送体系。做大棉袜、梅花鹿、黄牛等原产地市场，培育义乌小商品城等批发市场，活跃二手房、二手车交易。大力发展电子商务，培育新的消费增长点。支持企业开拓海外市场，外贸进出口增长高于全省平均水平。全面开展第三次经济普查，为经济协调健康发展提供详实依据。

　　（二）加快产业转型升级。坚持分类指导、扶优扶强、集群发展，着力构建现代产业体系。发展重点产业。发挥资源优势，依托骨干企业，集中各类要素，重点打造装备制造和农产品深加工两大主导产业，做大做强高精铝加工、纺织袜业、医药等特色优势产业。积极培育新材料、新能源、信息软件等战略性新兴产业。完善区域配套功能，推进商圈建设，加快发展金融保险、商贸物流等生产性服务业和家政、养老、健康等生活性服务业。开发特色文化资源，打造精品旅游线路，制定实施旅游业发展规划，推动文化旅游产业融合发展。培育骨干企业。大力发展民营经济，建立负面清单制度，引导民间资本投入实体经济，进入基础产业、基础设施和公用事业等更多领域。积极争创全省民营经济综合改革示范区。支持骨干企业技术改造、更新产品结构，实现高端化、规模化、集群化发展。加快小微企业培育孵化和中小企业晋档升级，培育小微企业400户，新增四上企业30户。建设现代园区。赋予开发区更多经济社会管理权限，推进一区两园深度融合，加快晋升国家级开发区步伐，带动龙山区、西安区产业升级。争取东丰、东辽工业集中区晋升为省级开发区。规划建设以金州为核心区域的生态产业开发区。打造服务平台。实施人才开发计划，积极引进专家型、候鸟型人才，培养技能型、实用型人才，鼓励辽源籍高校毕业生回乡创业就业。深化与金融机构的战略合作，加强金融生态环境治理，搭建金融资产交易、物权登记托管和债券融资增信平台，鼓励发展企业担保机构，加快重点企业上市步伐。强化中小企业服务联盟，启动运行创业服务大厦。

　　（三）全面深化改革开放。把改革创新贯穿于经济社会发展各领域、各环节，不断创新体制机制，增强发展活力动力。推进行政体制改革。转变政府职能，充分发挥市场配置资源的决定性作用，切实强化政府宏观调控、市场监管、社会管理、公共服务职能。继续清理行政审批事项，下放微观管理职能。完成事业单位分类改革和卫计委、食药监等机构改革。组建行政服务中心和公共资源交易中心。推进社会事业改革。鼓励社会力量办学，促进教育布局调整。完善公立医院制度，加快基层医疗卫生机构综合改革。推动养老服务改革。探索政府购买公共服务。推进城镇化配套改革。进一步放开户籍制度，加快双向一体化发展。完善节约集约利用土地机制，盘活和有效利用存量土地，积极争取列入省建立城乡统一建设用地市场试点和农村宅基地流转试点。推进财税体制改革。扩大营改增试点范围。完善国有资产管理办法。加强政府债务管理，实现举债适度、程序严格、管理规范、运行高效、风险可控。推进金融创新。支持金融企业创新金融产品，发展手机金融和网络金融。探索建立粮食银行。力争土地收益保障贷款试点全覆盖。创新推动林权抵押贷款。推进生态文明制度体系建设。划定生态保护红线，实行最严格的源头保护、损害赔偿和责任追究制度。完善生态资产产权和用途管制制度、生态补偿制度和生态文明考评办法。推进招商引资跃上更高层次。围绕打造主导产业、特色产业，下气力引进一批战略投资者。更加注重发挥已落户外埠企业和本地企业作用，更加注重项目甄别和合同约束，进一步浓厚招商氛围，切实提高招商实效。争取签约落地超亿元项目50个以上，其中超10亿元项目20个以上，引进域外资金515亿元，实际利用外资2.8亿美元。

　　（四）统筹推进城镇化。按照试点先行、梯次推进的总体安排，提升城乡综合承载功能，统筹推进具有辽源特色的新型城镇化。强化规划先导作用。修订完善南部新城建设规划，启动实施北部组团建设规划，编制外环路两侧控详规、市容市貌提升改造规划、城市路网规划，稳步实施新城区开发和老城区改造，不断优化城市空间结构和管理格局。完善城镇规划体系，提升东丰、东辽县城承载功能，加快金州省级试点镇建设，积极推动那丹伯、横道河、拉拉河、渭津、建安等重点镇产城融合发展。加快推进基础建设。伊开高速一期建成通车，加快辽长铁路建设，做好伊开高速二期、集双高速、长辽城际铁路、四松铁路项目前期工作。统筹推进引松入辽和东辽河干流城防工程，继续改造城市供排水系统。实施八路一桥工程，维修20条巷路。完成北部热源厂建设。促进城市管理升级。协调推进国家级卫生城、园林城、文明城和环保模范城创建工作，力争国家级园林城达到申报条件。加强城市景观建设，普及市树、市花，植入现代时尚元素，增强城市魅力活力。开展市容环境综合整治，建立城市管理长效机制。强力拆除违法建筑，合力推动房屋征收，更加注重依靠法治手段和群众力量解决重点难点问题。积极打造智慧城市。立足信息化建设成果普惠百姓，启动城市公共信息平台、智慧医疗、智慧教育、智慧气象、云存储等应用项目，完善中小企业服务平台、电子商务、智慧城管等运行系统。实现市民卡发放全覆盖。加快推进软件园区建设。组建智慧城市产业发展集团。

　　（五）积极支持县域发展。建立统筹规划、区域协调、资源共享、优势互补的新型市县关系，推动以工促农、以城带乡、工农互惠、城乡一体发展。壮大县域实力。以工业化引领和推动农业产业化，培育壮大特色主导产业，构建县域产业支撑体系，推动县域经济板块做大做强。支持鑫达钢铁、金刚水泥等重点企业转型升级，实现可持续发展。引导睿康、鹏翔、夏兴等龙头企业延伸产业链条，提高加工深度和高端产品、终端产品比重。支持麒鸣牧业确立中国高端羊肉品牌地位。力争把金翼集团打造成蛋制品行业的双汇和生物质能源循环利用的全国样板。促进农业增效。粮食产量稳定在40亿斤阶段性水平。新改扩建蔬菜棚室1000栋，新建标准化牧业小区30个。做大果品、梅花鹿、柞蚕、食用菌等特色产业。加快建设拉拉河聚鲜园蔬菜产业园、南屯基秀水现代农业观光园，打造无公害绿色有机农产品生产基地。积极推进农业科技创新，支持农科院技术研发，带动高效农业发展。创新农业经营方式。扩大农村集体土地所有权确权登记颁证试点范围，农村承包地流转面积增长10%以上。发展家庭农场、农业企业200个以上，新增农民专业合作社80个以上。鼓励引导工商资本下乡。多渠道、宽领域增加农民财产性收入。

　　（六）深入实施民生工程。以保基本、兜底线、促公平、可持续为准则，继续推进惠民实事行动计划，让改革发展成果更多更公平地惠及全市人民。实施住房保障工程。保障房在建规模210万平方米，回迁居民1.2万户。综合整治7个住宅小区。力争完成剩余的暖房子改造计划。推动弃管小区物业管理全覆盖。推进创业就业工程。扶持3000人成功创业。建设3个省级高质量就业示范社区。新增城镇就业3.5万人。农村劳动力转移就业16.5万人。完善社会保障体系。继续提高养老保险待遇标准。制定促进非公经济组织和小微企业参保政策。有效解决国企改制失业金发放和厂办大集体改革相关问题。开展困难群体救助。进一步提高城乡低保标准。完善城镇居民大病救助政策。开展残疾人康复服务3500人次。实施弱势群体100%法律援助。全面启动贫困家庭学生资助计划。加快养老机构建设。制定养老服务业发展规划。建立社区养老服务网络。开展政府购买养老服务试点。探索社会化、市场化、专业化养老服务模式，鼓励发展高端养老产业。积极推进智慧养老项目。办好人民满意教育。按需整合教育资源，推进教育均衡发展。筹建职业教育集团，扩大国内外教育合作。深入实施素质教育，加强德育、体育教育，大力整治师德师风。改善医疗计生服务。整合医疗卫生资源，改善医疗服务条件。建设健康教育主题公园。完成130个村卫生室标准化建设。配备32台乡镇120急救车。为城乡65岁以上老年人免费体检。实施单独两孩生育政策。提升公共服务水平。新增管道燃气用户1万户。城区24小时供水率达到85%。大力推进城市公交公车公营。推进文化惠民。完善公共文化服务体系，为群众性文化活动提供便利和支持。全力办好十七届省运会，更新一批全民健身设施。建设数字图书馆、数字档案馆。开发保护特色历史文化资源。

　　（七）切实推进生态建设。坚持在发展中保护，在保护中开发，努力建设天蓝地绿水净的美丽辽源。着力加强大气污染防治。推进10蒸吨以下小锅炉改造，实施7个工业减排项目、60个农业减排项目，加快推进鑫达钢铁、大唐电厂、巨峰生化等重点企业脱硫脱硝减排，加强国控重点污染源企业在线监控。清理整治市区露天明火烧烤。支持发展生物质能源，严禁农村秸秆焚烧。着力加强水污染防治。推进15个东辽河流域水污染防治项目，实现东辽河出境断面水质稳定达标。实施杨木水库水源地一、二级保护区全面禁排，加快推进人工湿地项目。深入开展农村饮水安全工程。启动实施仙人河（半截河）综合治理和分流工程。着力加强生态修复治理。实施沉陷区内居民、企业整体搬迁，彻底清理煤场、矸石场，推进矿山湿地公园绿化和景观建设。深入实施第二个十年绿美规划，加强林地清收保护，严厉打击毁林开荒，市直属国有林场实行禁伐，区属生产生活用林按需限伐。启动农村人居环境改善工程，争创国家级、省级生态村镇。

　　（八）创新社会治理体系。强化源头控制、矛盾调处和依法治理，实现政府治理和社会自我调节、居民自治良性互动。推进依法治市，深入开展六五普法，充分发挥四个基地、法律咨询服务平台作用。严格落实重大决策社会风险评估机制，建立健全畅通有序的诉求表达、纠纷调处、权益保障机制。推进平安辽源建设，形成立体化、智能型、全覆盖的社会治安防控体系。加大社区建设投入，完善社区基本功能，积极推进网格化管理和社会化服务。进一步巩固民族团结、宗教和顺良好局面。健全完善食品药品安全监管体系，推进食品药品放心工程。完善防灾减灾机制，加强突发事件应急管理。深入推进安全发展。坚持预防为主、标本兼治原则，严格落实安全生产主体责任、属地责任、监管责任，加强责任倒查和责任追究。推进安全生产标准化建设和网格化监管，建立健全隐患排查长效机制。

　　三、加强政府自身建设

　　以开展党的群众路线教育实践活动为契机，创新和加强政府服务与管理，致力建设人民满意政府。

　　一是强化执行力。认真学习贯彻习近平总书记一系列重要讲话精神，把学习成果转化为推动发展、造福民生的实际成效。坚决落实省委、省政府和市委决策部署，自觉接受市人大的法律监督、工作监督和市政协的民主监督。坚持依法行政，注重运用法治思维和法治方式解决实际问题，切实维护社会公平正义。加强能力建设，提高办事效率，始终勤勉尽责、毫不懈怠，努力把辽源各项事业不断推向深入。

　　二是强化创新力。不断解放思想，增强开拓精神和进取意识，勇于担当，攻坚克难，提高工作主动性、创造性。以创新促进改革深化和突破，努力破除体制机制弊端，最大限度凝聚改革正能量、释放改革红利。以创新促进政府工作提效、干部作风转变，构建务实高效的行政管理体制。以创新推动科学发展，让发展更有效率、更可持续，让百姓生活得更加幸福、更有尊严。

　　三是强化公信力。严格落实八项规定，坚决纠正四风一顽症。坚持为民务实干事，倾听群众呼声，牵挂百姓冷暖，密切同人民群众的血肉联系。坚持接地气、重行动，争做解放思想的表率、深化改革的先锋和狠抓落实的楷模。大力加强软环境建设，严肃整治不作为、乱作为问题。厉行勤俭节约，提倡艰苦奋斗，把有限资源更多地向发展和民生倾斜。坚持廉洁从政，从严治政，认真落实党风廉政建设责任制，健全民主决策、风险防控和权力运行监督机制，推进惩治和预防腐败体系建设。严肃查处各类违法违纪案件，着力解决群众身边的腐败问题。推进政府信息公开，保障群众的知情权、参与权、监督权，让人民监督权力，让权力在阳光下运行。

　　各位代表，辽源正处在深化改革、全面转型的关键时期，我们的任务艰巨繁重、使命光荣神圣。让我们在市委坚强领导下，解放思想，振奋精神，团结拼搏，扎实工作，为推动我市转型发展、全面振兴，为建设幸福辽源、美丽辽源而努力奋斗！
